# The Relation of the Physician and the Druggist

**Published:** 1906-02

**Authors:** F. S. White

**Affiliations:** Terrell, Texas


For Texas Medical Journal.

### The Relation of the Physician and the Druggist.

BY DR. F. S. WHITE, TERRELL, TEXAS.

All professions and avocations in life are more or less dependent upon other professions and avocations. I will not take up your

*Read before the Kaufman County Medical Society, December 5, 1905.

time in elaborating this idea, but will only say that this is particularly true of the medical profession and the druggist or pharmacist. The very nature of our calling and the manner of practicing the same emphasizes the closeness of this relationship. At the present time the manufacturing pharmacists are very important factors in the practice of medicine. They place before us many and various ready-made and eligible preparations, and insist upon us prescribing them in original packages. This most of us do to such an extent that nearly all of our prescriptions contain one or more of these proprietary combinations. They are more often used as a matter of convenience than because they are just what we want, but either on account of laziness or hurry we make a prescription for one of these much-recommended and advertised combinations, rather than to formulate a mixture that meets our wants and the indications of the case being treated. This tendency on the part of the medical profession is gradually making simply clerks out of the local druggists where they should be scientific pharmacists. We depend upon the manufacturers to tell us what preparations are incompatible and what are good for certain diseases. The tendency to allow the manufacturer to do our thinking and prescribing is reaching a point when it seems that about all that is necessary is to keep well up with the new remedies and the old combinations ready-made to order. I believe it is about like buying a ready-made suit of clothes, because it takes less time, and is cheaper than to have a suit made that exactly fits. It takes less work to write a prescription for ready-made articles than to work out a formula that exactly fits the case under consideration. Now, don't understand me as condemning all proprietary medicine. I believe that many of them are very eligible and effective. What I do criticise is: physicians prescribing them without the proper regard for what they contain, and for the exact indications of the case. This practice begets in the physician habits of carelessness and disregard for details. Our success depends largely upon our attention to the little things and the details connected with our work. If a physician thoughtfully formulates a prescription for a given case, he is much more apt to prescribe just what is needed than if he simply writes for a proprietary combination that contains a little of everything and not much of anything. Most of the preparations look good and taste good, hence from that standpoint are very satisfactory to our patients who do not know what the medicine contains, or whether or not it is what they need. I have no objection to prescribing proprietary medicines on principal. My contention is that on account of convenience and eligibility we very often are satisfied with something which we recognize is not just what we want to give.

I now come to another and much more important phase of this subject. On account of, and as a natural sequence of our prescribing ready-made preparations, our patrons soon learn that for certain conditions we prescribe certain remedies, and, as a result, when they make their own diagnosis, they go to the druggist and call for certain things. They do this in order to save the expense of paying the doctor for a prescription. In this way we have built up and created a great demand for some preparations which are capable of doing much harm when taken indiscriminately. This also encourages and justifies the druggist in prescribing for our patients. It is a well known fact that many druggists regularly treat cases of sickness which should come to the physician. Some druggists have a very extensive practice, especially in the treatment of venereal diseases. They keep certain preparations which they give for gonorrhea, chancroid, etc., whether they are indicated or not. Let a man go to a certain druggist and tell him that he has the "clap," and he will get an injection and medicine to take and be given instructions to call at regular intervals. They will also treat children and adults for malaria and bowel trouble, and almost everything else from an ingrowing toenail to meningitis. They will treat colicy babies and babies with all sorts of diarrheas, coughs and colds. They have the remedies which are recommended for all these conditions and are guaranteed to cure them, and they proceed to sell them indiscriminately. Now, this is a practice on the part of the druggists which I absolutely condemn. The function of the druggist is to fill prescriptions, and to sell their stock when called for. They have no license to practice medicine, and are not qualified to prescribe for sick people, and should be prosecuted for doing so. If a person comes into a drug store and calls for a certain medicine, there is no objection to selling it, but if a person comes into a drug store and wants something for a certain diseased condition, the druggist has no right under our laws to prescribe and sell anything to him. He should be told to consult a physician whose business it is to treat the sick.

There is another phase of this question: The most of us send our prescriptions to the druggists to be filled,—we do not infringe upon them by keeping our medicine and selling it to our patients; therefore, they have no right to infringe upon us by prescribing for sick people.

Physicians, as a rule, are the most patient and long-suffering set of men to be found in any community. We generally submit to unjust imposition upon the infringement of our rights and prerogatives. We have it in our power to absolutely stop the druggist from prescribing for sick people, and thus imposing upon the people, and taking from us what rightfully belongs to us, besides lowering the medical profession in the estimation of the public. Many of the people are led to believe that an old druggist knows about as well how to treat diseases as physicians. All of us have seen the terrible result of the treatment of diseases by druggists. Innumerable numbers of men are now the victims of chronic and incurable conditions as a result of being treated for venereal diseases by druggists. Especially does this apply to gonorrhea. Many babies are annually sent to untimely graves on account of this nefarious practice, and often the doctor is severely criticised for not saving the baby when the die was cast by the druggist before the physician saw the case. We should absolutely refuse to allow our prescriptions to go to a druggist who so flagrantly disregards our interest and the interest of the public. By this means we can remedy to a very large extent this growing evil.

The manufacturing chemists load our offices with samples, and insist upon our prescribing their particular brand of certain remedies. They often go so far as to leave with us ready-printed prescriptions for certain products,—all we have to do is to sign our names and hand to the patient. This free-sample evil robs us of many patrons and dollars. We often give these samples to the sick. They see what it is and what it is recommended for, and they say it has the sanction of the family physician, so in the future when any of their family or friends get sick, they immediately refer them to a certain proprietary medicine, and say that Doctor So and So" prescribed it. And as a result the proprietary manufacturer profits by our losses, and the people take many things not suited to their particular case. This practice has grown until it is almost as great a menace to the physicians as the patent medicine evil. A large number of such preparations contain a considerable quantity of some derivative of opium, and often their continued use makes opium habitues for which the doctor is largely responsible.

In reality the true relation between the physician and the druggist is very intimate and confidential. We all, at some time in our lives, under stress of hurry or overwork, make dangerous mistakes in our prescriptions. If the druggist is what he ought to be, he detects these errors, and protects the patient and the physician without detriment to either.

We all go to the druggist for information as to compatibles, etc. Very often we place confidence in him, and the druggist who would betray that confidence is devoid of every principle of honor and manhood. I am proud to say that strictly unscrupulous druggists are very rare.

